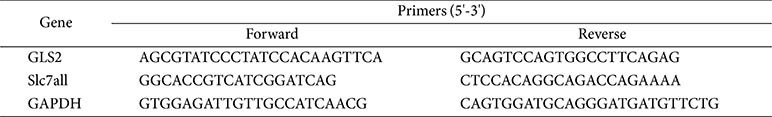# Untargeted Serum Metabolomic Profiling Reveals Metabolic Inflammation Induced by *Stutzerimonas stutzeri* PM101005 Isolated from Atmospheric Particulate Matter

**DOI:** 10.4014/jmb.2507.07027

**Published:** 2025-11-04

**Authors:** Subin Park, Jun-Young Park, Kyung-Soo Lee, Yu-Jin Jeong, Chang-Ung Kim, Moo-Seung Lee

**Affiliations:** 1 Environmental Diseases Research Center, Korea Research Institute of Bioscience and Biotechnology, Daejeon 34141, Republic of Korea; 2 Department of Biomolecular Science, KRIBB School of Bioscience, Korea University of Science and Technology (UST), Daejeon 34113, Republic of Korea; 3 Research Institute of Pharmaceutical Sciences (RIPS), College of Pharmacy, Chosun University, Gwangju 61452, Republic of Korea

**Keywords:** Particulate matter, *Stutzerimonas stutzeri* PM101005 (PMSS), metabolomics, inflammation

## Abstract

Particulate matter (PM), a major pollutant of air pollution, contains a complex mixture of chemical and biological elements that pose significant threats to human health. Among the biological components, *Stutzerimonas stutzeri* PM101005 (PMSS), a bacterium isolated from fine dust, has been identified as a contributor to respiratory damage through inflammation. However, the mechanisms underlying its pathogenicity, particularly in comparison to environmental strains such as *S. stutzeri* (SS), remain unclear. In this study, we aimed to investigate the systemic effects of PMSS by comparing the serum metabolite profiles and inflammatory responses induced by SS and PMSS infections in a mouse model. Mice infected with PMSS exhibited marked alterations in serum metabolites, many of which were associated with enhanced pro-inflammatory signaling and the suppression of anti-inflammatory pathways. These metabolic changes were accompanied by elevated levels of circulating inflammatory cytokines, indicating a link between PMSS infection, metabolic dysregulation, and systemic inflammation. Our findings demonstrate that PMSS-associated bacterium, induces inflammation through modulation of host serum metabolites. This study suggests that PM-induced changes in serum metabolites contribute to inflammation, highlighting the need for further research on the systemic effects of biologically active components within particulate matter.

## Introduction

Particulate matter (PM) is a complex mixture of solid and liquid particles, the size, chemical composition, and other physical and biological properties of which are determined by location, time, climate, and season [1]. Many studies have shown that PM can have acute and chronic health effects on humans, negatively affecting various systems and organs, including the respiratory, cardiovascular, digestive, nervous, immune, and reproductive systems [2]. In particular, exposure to PM has negative effects on respiratory diseases, including increased lung inflammation and worsening respiratory symptoms [3]. *Staphylococcus* sp., *Haemophilus influenzae*, *Pseudomonas aeruginosa*, *Streptococcus* sp., *Streptococcus pneumoniae* and *Aspergillus fumigatus* are common respiratory bacterial pathogens that cause allergies and respiratory diseases [4, 5].

*Stutzerimonas stutzeri* is widely distributed in the environment, including straw, manure, soil, and canal water, and has been isolated from humans as an opportunistic pathogen. It is generally mobile, rod-shaped, nonfluorescent denitrifying bacteria [6]. *S. stutzeri* present in PM (PMSS) induces lung damage *in vivo* through more severe inflammatory responses, infectivity, and cytotoxicity than the SS environmental strain [4]. However, the molecular and metabolic mechanisms underlying the enhanced pathogenicity of PMSS remain poorly understood, underscoring the need for further mechanistic investigation.

Previous studies have shown that *S. stutzeri* PMSS from particulate matter induces stronger inflammation and lung damage compared with standard strains. It activates immune responses through TLR2/4/5 and NF-κB/MAPK pathways. These findings suggest any potential bacterial risk in air pollution-related lung injury. The studies have shown that PMSS induces more severe inflammatory responses, infectivity, and cytotoxicity than SS, but the differences that lead to different patterns in infection have not been addressed. Therefore, we focused on the need for further research into the differences between SS and PMSS, and approached the viewpoint that there would be differences in metabolites. Microbiological, immunological, and toxic agents are known to initiate inflammatory responses by activating a range of humoral and cellular mediators. Recent studies have suggested that metabolic enzymes, intermediates, and regulatory molecules play active roles in modulating inflammatory signaling pathways [7]. Furthermore, systemic inflammatory diseases such as rheumatoid arthritis (RA) have been associated with profound metabolic alterations, suggesting a strong interconnection between inflammation and metabolism [8].

In the present study, we investigated whether infection with PMSS leads to distinct metabolic alterations compared to infection with SS. Given the known capacity of airborne pollutants to influence host immune responses, and the emerging evidence that microbial components within PM can enhance pro-inflammatory signaling, we hypothesized that metabolites upregulated during PMSS infection would be associated with metabolic pathways involved in inflammation. To address this, we conducted untargeted metabolomic profiling of serum from mice infected with SS or PMSS. Our results confirmed that both SS and PMSS infections induce inflammatory responses, with PMSS triggering a significantly more pronounced effect. To further evaluate their biological relevance, we treated MH-S alveolar macrophages with these metabolites and observed a marked upregulation of inflammatory markers, supporting their potential role in mediating PMSS-induced inflammatory responses.

These findings suggest that PMSS infection alters host serum metabolite profiles in a manner that promotes inflammation. This study provides new insight into the metabolic basis of the heightened inflammatory response induced by PMSS and highlights the role of airborne microbial pollutants in modulating host-pathogen interactions through metabolic pathways. Ultimately, these results improve our understanding of why SS and PMSS elicit different infection outcomes, and how specific metabolites contribute to inflammation in the context of PM-associated bacterial infection.

## Materials and Methods

### Mice

Wild-type (WT) C57BL/24 mice (8 weeks old) were purchased from Koatech (Republic of Korea). All animal experiments were approved by the Institutional Animal Care and Use Committee of the Korea Research Institute of Bioscience and Biotechnology (Republic of Korea) (Approval No. KRIBB-ACE-21193).

### Preparation of Bacteria and Infection *in vivo*

In this study, PMSS (GenBank: CP046902.1) isolated from ERM-CZ100 and SS (ATCC 17588) were used. Each bacterial colony incubated in Luria-Bertani (LB) medium was inoculated into 5 ml of LB medium and cultured overnight at 37°C with continuous shaking at 200 rpm. The culture suspension was diluted 1:5, transferred to fresh medium, and incubated with shaking at 37°C for 2 more h. Bacterial cells were harvested by centrifugation at 10,000 ×*g* for 20 min. The cell pellets were washed with phosphate-buffered saline (PBS; pH 7.4) and then resuspended in sterile PBS to a final concentration of 1 × 10^8^ colony-forming units (CFU) per milliliter. For mouse infections, both SS and PMSS strains were prepared by washing and adjusting in sterile PBS to the desired concentration, and each mouse received an intranasal (i.n.) inoculation containing 3 × 10^8^ CFU. The optical density (OD) of the bacterial suspension was determined at 600 nm using a Spectra MAX 190 Microplate Reader (Molecular Devices, USA). Based on the OD readings, bacterial cultures were diluted accordingly to obtain the required concentrations for subsequent experimental procedures.

### Cell culture and Bacterial Infection

MH-S cells were cultured in RPMI 1640 medium (Corning, Thermo Fisher Scientific, USA) containing 10%FBS (HyClone, USA) and supplemented with 5.0 μg/ml streptomycin and 5.0 U/ml penicillin. MH-S cells were seeded in 12-well plates at a density of 2 × 10^5^ cells/well and infected with SS or PMSS at a multiplicity of infection (MOI) of 1/10 for 60 min. Extracellular bacterial growth was inhibited by treatment with gentamicin (50 μg/ml). Culture supernatants and cell lysates were collected at the indicated time points post-infection for further analysis.

### Untargeted Metabolomics Analysis (LC-MS/MS)

Untargeted metabolomics analysis of serum samples was performed using Nano LC coupled with a Q-Exactive mass spectrometer. Six samples were analyzed once in both positive and negative ionization modes. Metabolites were extracted by adding 100 μl of 100% methanol to 20 μl of serum, vortexing for 1 min, and incubating at 20°C for 1 h. Samples were centrifuged at 14,000 ×*g* for 10 min, and the supernatant was transferred to a new tube, dried using a speed-vac, reconstituted in 5 mM ammonium acetate, and filtered through a 0.22 μm spin filter. A 1 μl aliquot was injected for LC-MS/MS analysis. Raw data (*.raw) were processed using Compound Discoverer version 3.3 (Thermo Fisher Scientific) with the “Untargeted Metabolomics with Statistics Detect Unknowns with ID using Online Database and mzLogic” workflow. Compounds were identified by spectral similarity searches against the mzCloud (ddMS2) and ChemSpider (formula- or exact mass-based) databases. Features detected in blanks were excluded. Metabolite annotations followed the Metabolomics Standards Initiative (MSI) guidelines: Level 2 identifications required <10 ppm mass error and mzCloud scores >80, whereas Level 3 identifications were based on ChemSpider matches with <5 ppm mass error. Redundant features were removed by comparing peak areas to retain unique metabolites. For bacterial metabolite profiling, the LB sample served as the blank. Individual searches of SS and PMSS samples were performed, and metabolites were retained only if their peak area was less than fivefold that of the blank and the mzCloud Best Match score was ≤80.

### Enrichment Analysis

Pathways containing metabolites that showed significant changes after SS/PMSS infection were identified using Metabo Analyst 6.0. Enrichment analysis was selected and data converted to KEGG ID was entered. Input Type was selected as DB type, Feature Type was selected as Metabolites, and then submitted. Then, KEGG was selected in Progress and submitted.

### Cytokine Measurement

Experimental groups consisted of PBS-treated controls, mice infected with the environmental strain SS (ATCC 17588), and mice infected with PMSS (PM101005). SS was used as the reference strain to assess whether PMSS exhibits enhanced inflammatory properties due to its origin from particulate matter. Time points for serum sample collection (1 and 2 days post-infection) were selected based on previous findings indicating that peak inflammatory cytokine levels within 24–48 h following intranasal exposure to PMSS (Jeong *et al*., 2023). After infecting WT mice with SS and PMSS at 3 × 10^8^ CFU each, the concentrations of TNF-α, IL-6, MCP-1, MIP-1α, and IL-1β in the serum were analyzed. Cytokine secretion was measured using ELISA kits (Invitrogen).

### WST Cell Viability Assay

MH-S cells (1 × 10^4^ cells per well) were seeded in 96-well plates and allowed to attach for 24 h. After incubation, the cells were treated with various concentrations of 0–0.1 mM glutamate for 24 h. Subsequently, 10 μl of the Quanti-MAX WST-8 Cell Viability Assay reagent (#QM5000, Biomax, Republic of Korea) was added to each well and the plates were incubated at 37°C for 1 h. The absorbance was then measured at 450 nm using a Microplate Reader (MicroDigital, Republic of Korea).

### Metabolite Treatment

Glutamate was purchased from Sigma-Aldrich, Inc. (USA). MH-S cells were seeded in 12-well plates at a concentration of 2 × 10^5^ cells/well and cultured in RPMI 1640 medium (Corning, Thermo Fisher Scientific) containing 10% FBS (HyClone, USA) and supplemented with 5.0 μg/ml streptomycin and 5.0 U/ml penicillin. After 24 h of incubation, the culture medium was replaced with serum- and antibiotic-free RPMI 1640 medium, followed by treatment with glutamate at final concentrations of 0, 0.1, 0.5, and 1 mM. Culture supernatants were collected after 18 and 24 h for further analysis.

### Transcriptome Analysis

Total RNA was extracted using a NucleoSpin RNA Plus column (Macherey-Nagel, Germany), reverse transcribed into cDNA using reverse transcriptase, and amplified using the NanoHelix RT-qPCR kit (NanoHelix, Republic of Korea). The PCR cycling was carried out under the following thermal cycling protocol: an initial incubation at 50°C for 2 min, followed by a denaturation step at 95°C for 10 min to activate the DNA polymerase. This was succeeded by 40 amplification cycles, each consisting of denaturation at 95°C for 15 sec, primer annealing at 60°C for 30 sec, and extension at 72°C for 30 sec. Results were quantified using SYBR Green technology, and data were analyzed using Light Cycler 96 System Analysis Software (Roche Diagnostics GmbH, Germany). The relative expression of each gene was normalized to GAPDH expression in the same sample. Primers used for real-time PCR are listed in Table 4.

### Statistical Analysis

Statistical analyses were conducted using GraphPad Prism version 5 (GraphPad Software, USA). Comparisons between two groups were assessed using a two-tailed Student’s *t*-test, while differences among multiple groups were evaluated by one-way analysis of variance (ANOVA), followed by post-hoc tests including Bonferroni’s and Tukey’s multiple comparison procedures. A *p*-value less than 0.05 was considered to indicate statistical significance.

## Results

### Number of Features Identified from Metabolomic Analysis of Mouse Serum after SS/PMSS Infection and Multivariate Analysis of Metabolomic Data

**Fig. 1:**
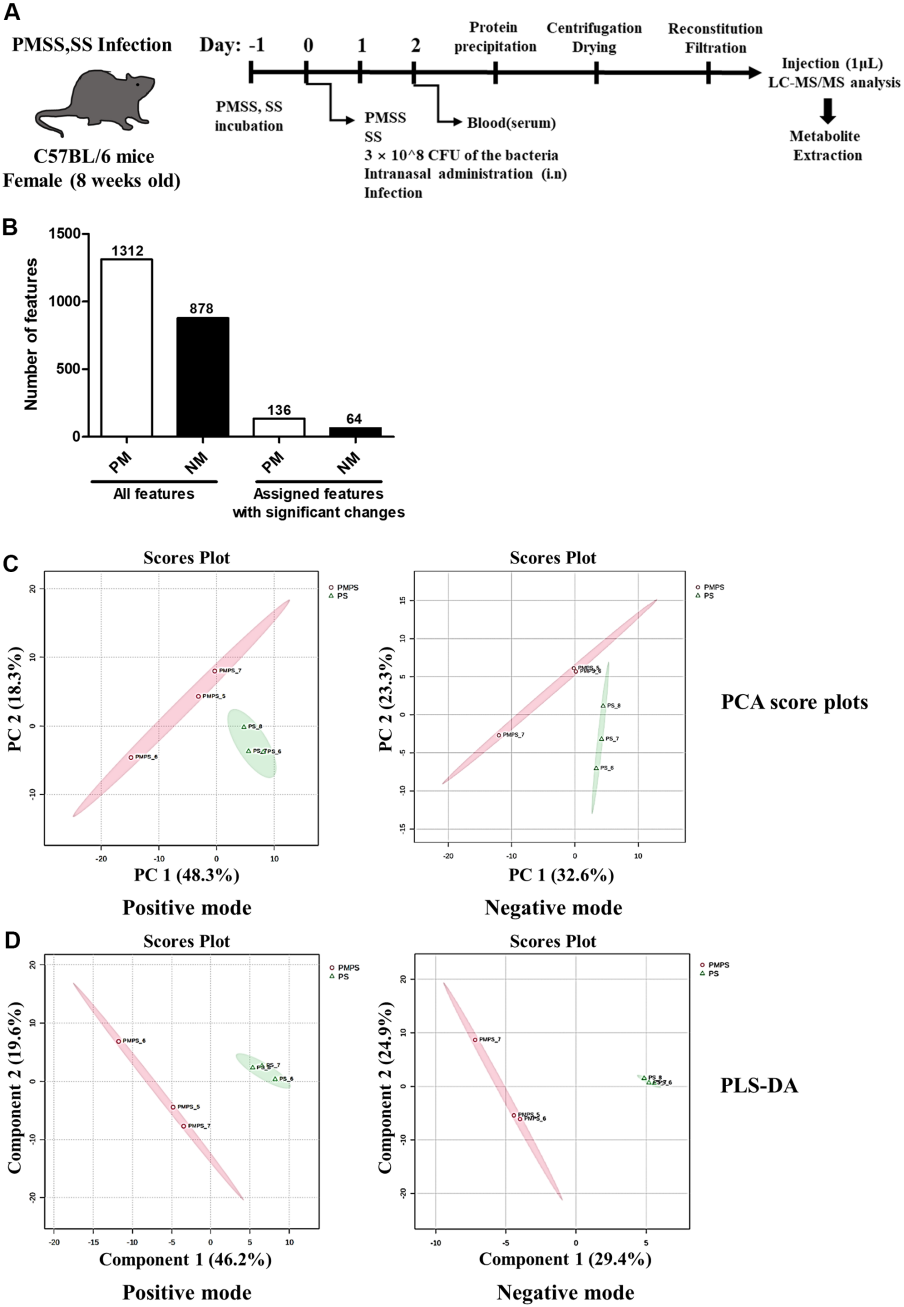
Metabolic feature distribution and multivariate analysis following SS/PMSS infection in mouse model. (**A**) Schematic overview of the experimental workflow. Female C57BL/6 mice (8 weeks old) were intranasally infected with 3 × 10^8^ CFU of either SS or PMSS strains. Serum samples were collected 24 h post-infection and subjected to metabolite extraction involving methanol precipitation, centrifugation, vacuum drying, and reconstitution in 5 mM ammonium acetate. Samples were filtered (0.22 μm) and 1 μl was injected for LC-MS/MS analysis. (**B**) Overview of detected metabolic features. Untargeted LC-MS profiling identified 1,312 features in positive ion mode (PM) and 878 features in negative ion mode (NM). Among these, 136 features in positive mode and 64 features in negative mode exhibited significant alterations in response to SS or PMSS infection compared to uninfected controls. (**C-D**) Multivariate statistical analyses. Principal component analysis (PCA) and partial least squares-discriminant analysis (PLS-DA) revealed clear metabolic separation between infected and control groups, highlighting distinct metabolic signatures induced by SS and PMSS infections.

Some microorganisms in PM cause allergic and respiratory diseases [4], and in particular, *Aspergillus fumigatus* induces the production of inflammatory factors in pulmonary microvascular endothelial cells (PMVEC) [9]. Given this, we sought to determine whether the up-regulated metabolites during PMSS infection were associated with metabolic pathways related to inflammation. Therefore, we first present the results of metabolite analysis in serum 2 days after infection with SS/PMSS to characterize metabolite changes in mouse serum infected with SS/PMSS (Fig. 1A). Of the 2,190 metabolic features, 1,312 positive mode features and 878 negative mode features were identified by LC-MS/MS analysis. Among them, 136 positive mode and 64 negative mode features showed significant change after infection with SS/PMSS strain (Fig. 1B). We visualized whether there were differences in metabolite levels between the SS-treat group and the PMSS-treat group in mouse serum infected with SS/PMSS using Principal Component Analysis (PCA) and Partial Least Squares Discriminant Analysis (PLS-DA) (Fig. 1C and 1D). Because there was a clear distinction between the SS treat group and the PMSS treat group, the two groups can be distinguished based on metabolites, suggesting that the difference in metabolite changes induced in mouse serum by SS/PMSS infection is likely to be statistically significant.

### Differentially Abundant Metabolite Analysis and the Effects of SS/PMSS Infection on Metabolite Composition

**Fig. 2:**
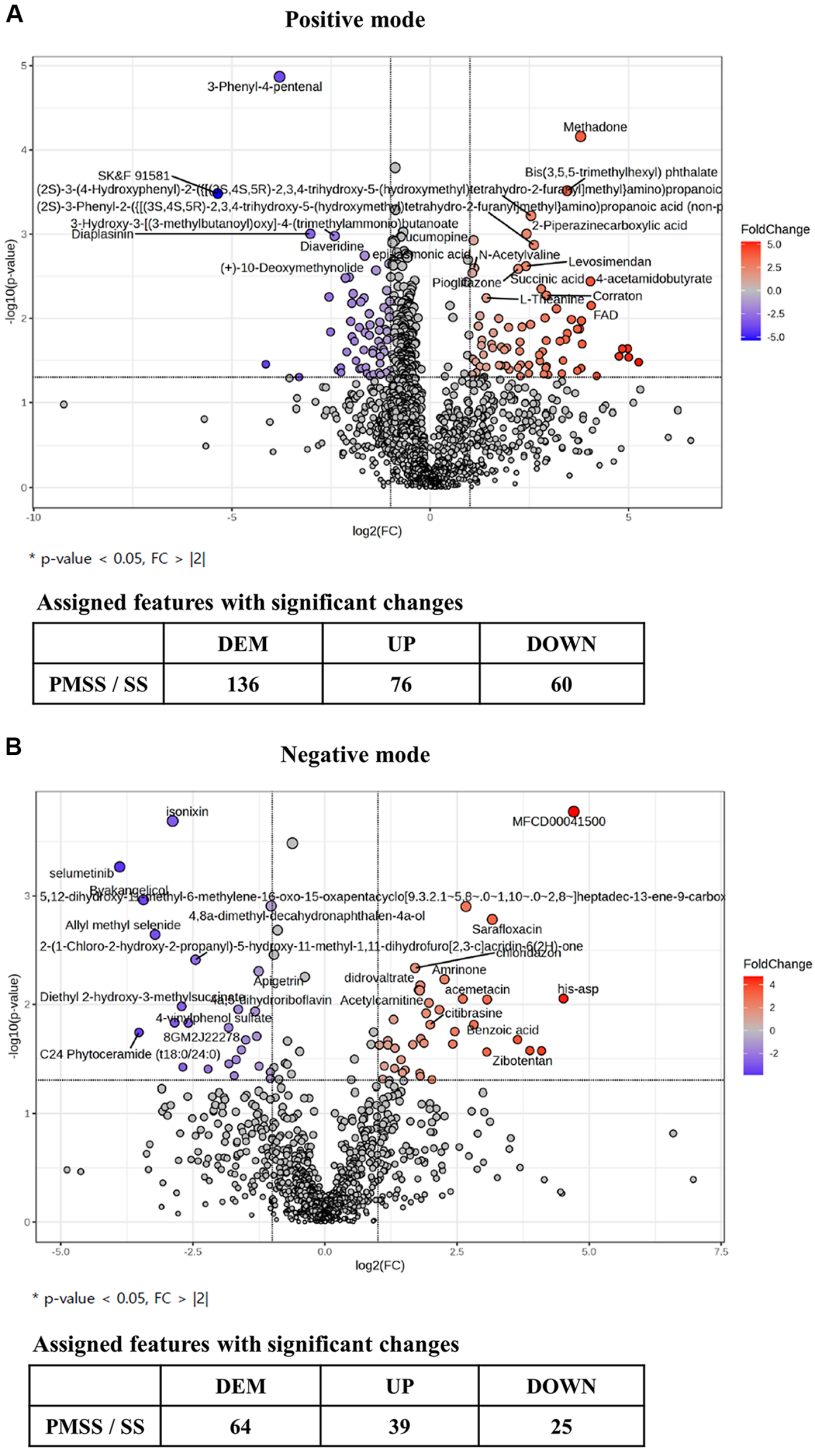
Volcano plots showing differentially regulated serum metabolites in SS- or PMSS-infected mice. Female C57BL/6 mice (8 weeks old) were intranasally infected with 3 × 10^8^ CFU of SS or PMSS bacterial strains. Serum metabolite profiles were analyzed using untargeted LC-MS. (**A**) In positive ion mode, volcano plot analysis identified 76 significantly upregulated and 60 downregulated metabolites following infection. (**B**) In negative ion mode, 39 metabolites were upregulated and 25 were downregulated. These results indicate widespread metabolic alterations induced by SS and PMSS infection. Metabolites were considered significantly altered based on a fold change (FC) > |2| and *p*-value < 0.05.

In Fig. 1C and 1D, PCA and partial least squares discriminant analysis (PLS-DA) demonstrated clear separation between the SS- and PMSS-infected groups, indicating substantial differences in secreted metabolite profiles in mouse serum. To further evaluate these differences, we performed untargeted metabolomic analysis in both positive and negative ionization modes. Secreted metabolites showing significant quantitative differences between the PMSS and SS groups were visualized using volcano plots (Fig. 2), based on statistical evaluation of Z-score–normalized data. For the selection of key metabolites, normalized quantitative values were subjected to statistical filtering. Metabolites that satisfied the criteria of *p* < 0.05 and |log2FC| > 1 were considered significantly altered between groups. In the positive mode, 76 metabolites were found to be up-regulated and 60 down-regulated, out of a total of 136 features. In the negative mode, 39 metabolites were up-regulated and 25 down-regulated, out of 64 features. Volcano plots illustrate the distribution of these metabolites according to statistical significance and fold change, thereby highlighting the differences in secreted metabolite composition between SS and PMSS infection.

To prioritize biologically relevant changes, the top 50 up-regulated and top 35 down-regulated secreted metabolites in SS/PMSS-infected mice, ranked by fold change and abundance, are presented in Tables 1 and 2, respectively. In addition to serum-derived metabolites, we further performed metabolite profiling of the bacterial cultures themselves. Table 3 summarizes the secreted metabolites derived directly from SS and PMSS, independent of host serum, thereby providing insight into strain-specific metabolite secretion patterns. Together, these analyses reveal distinct host- and pathogen-derived metabolite changes associated with SS and PMSS infection.

### Discriminatory Serum Metabolite Features Identified by Random Forest Modeling and Hierarchical Clustering in SS- or PMSS-Infected Mice

**Fig. 3:**
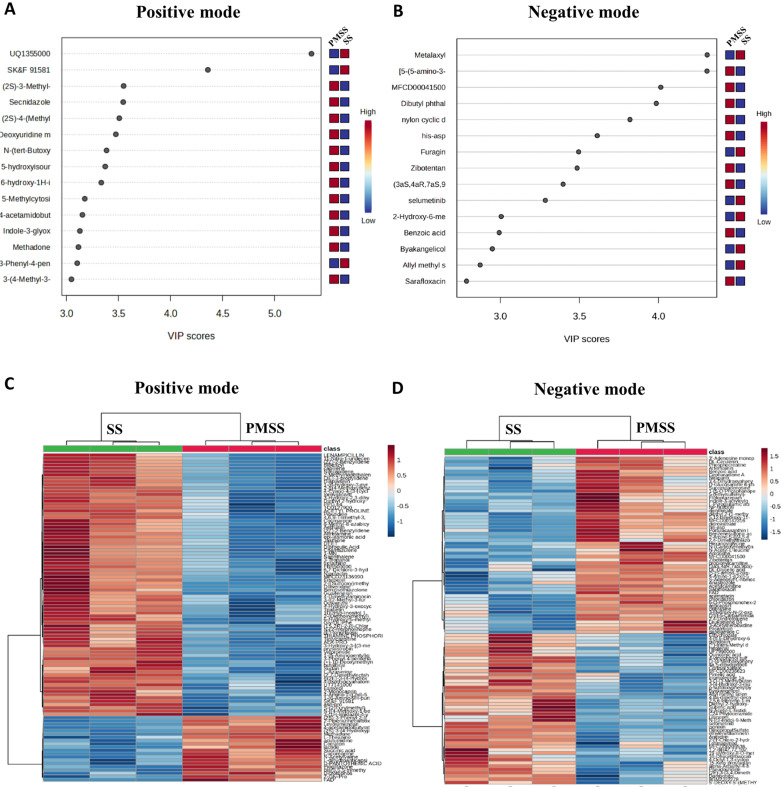
Discriminatory serum metabolite features identified by random forest modeling and hierarchical clustering in SS- or PMSS-infected mice. Female C57BL/6 mice (8 weeks old) were intranasally infected with 3 × 10^8^ CFU of SS or PMSS, and serum metabolites were profiled using untargeted LCMS. (**A–B**) Random forest modeling identified the top 15 metabolites with the greatest mean decrease in accuracy, highlighting their discriminatory power between infection and control groups. (**C–D**) Heatmaps of the top 100 significantly altered metabolites from both positive and negative ionization modes, generated using log2-transformed and z-score–normalized data, revealed distinct clustering patterns and clear separation between groups, indicating infection-driven metabolic reprogramming.

In Fig. 1D, we could confirm the metabolic differences between the SS, PMSS infection groups and the control group. To quantitatively evaluate the contribution of each metabolite to group discrimination, Variable Importance in Projection (VIP) scores were calculated. Metabolites with a VIP score greater than 1 were considered significant contributors to the observed differences [10]. The 15 most influential metabolites for distinguishing between the SS and PMSS groups were visualized based on their VIP scores. In the positive ion mode, 78 metabolites were significantly upregulated and 60 were downregulated in the infection groups (Fig. 3A). Similarly, in the negative ion mode, 39 upregulated and 25 downregulated metabolites were identified as significantly altered (Fig. 3B). Furthermore, the top 100 most upregulated and downregulated metabolites in both the SS and PMSS groups, relative to the control group, were visualized using a heatmap to highlight overall metabolic changes.

### Metabolite Set enrichment Analysis (MSEA) of Differentially Regulated Serum Metabolites in SS- or PMSS-Infected Mice

**Fig. 4:**
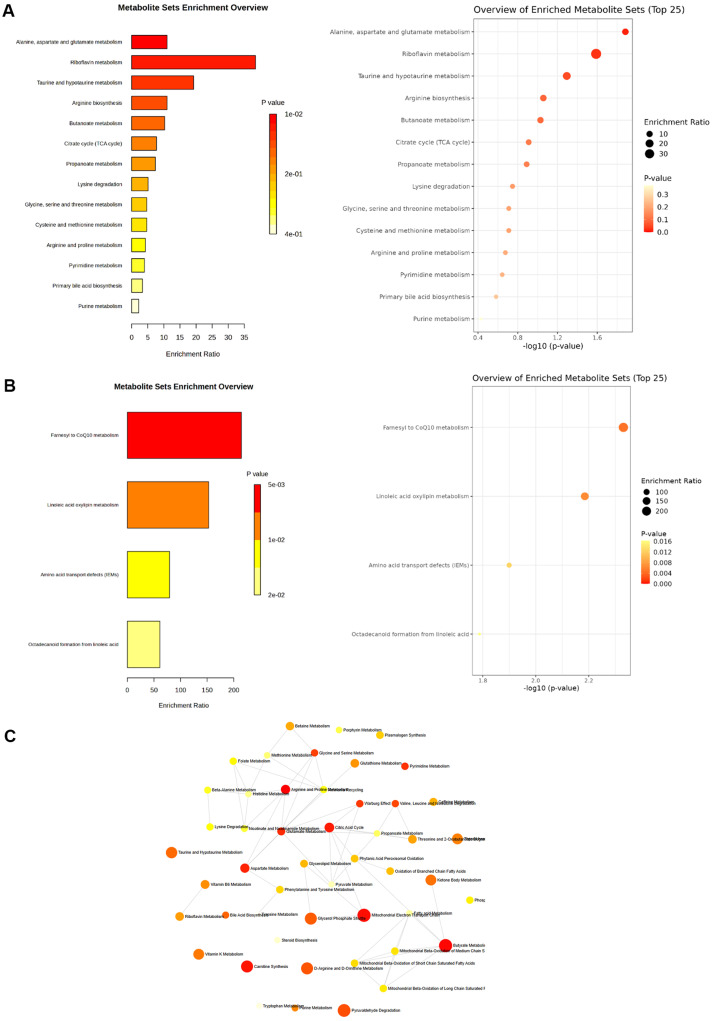
MSEA of differentially regulated serum metabolites in SS- or PMSS-infected mice. 8- week-old female C57BL/ 6 mice were intranasally infected with 3 × 10^8^ CFU of either SS or PMSS. Serum metabolites profiled by untargeted LC-MS were subjected to MSEA using significantly altered metabolites. (**A**) PMSS infection upregulated pathways related to alanine, aspartate and glutamate metabolism (**B**) PMSS infection downregulated pathways associated with Farnesyl to CoQ10 and linoleic acid oxylipin metabolism, suggesting targeted suppression of host metabolic processes. (**C**) Metabolite interaction network showing interconnections with amino acid and energy metabolism pathways.

By synthesizing the major metabolites identified in positive and negative modes, we visually represented the differences between SS and PMSS. In the PMSS infection group, MSEA was performed to identify the pathways involved in these metabolites. As a result of enrichment analysis, the metabolites up-regulated by PMSS infection were mainly involved in the metabolism related to alanine, aspartate, and glutamate, and were also involved in the activation of metabolism related to riboflavin and butanoate (Fig. 4A). Also, MSEA showed that the down-regulated metabolites were involved in the inhibition of farnesyl to CoQ10 and linoleic acid oxylipin metabolic pathways (Fig. 4B). Among the significantly enriched metabolic pathways identified in the MSEA, alanine, aspartate, and glutamate metabolism was ranked highest despite exhibiting a lower enrichment ratio compared to riboflavin metabolism. Among these, alanine, aspartate, and glutamate were found to play a role in enhancing the inflammatory response [11-13], while CoQ10 and linoleic acid oxylipin were found to contribute to the relief of inflammation and stress [14, 15]. Fig. 4C presents the metabolite interaction network for the top enriched pathways, highlighting alanine, aspartate, and glutamate metabolism as central nodes. Notably, glutamate metabolism exhibited strong interconnections with multiple metabolic pathways, including those of aspartate, citric acid, glycine, serine, arginine, and proline. This network suggests that perturbations in glutamate metabolism may serve as a key hub linking diverse amino acid and energy metabolism pathways. Through this, it was confirmed that PMSS infection mainly affects the metabolic pathway that induces the inflammatory response, and also affects various metabolic pathways that regulate the inflammatory response.

### Quantification of Pro-Inflammatory Cytokine Levels in SS- and PMSS-Infected Mice

**Fig. 5:**
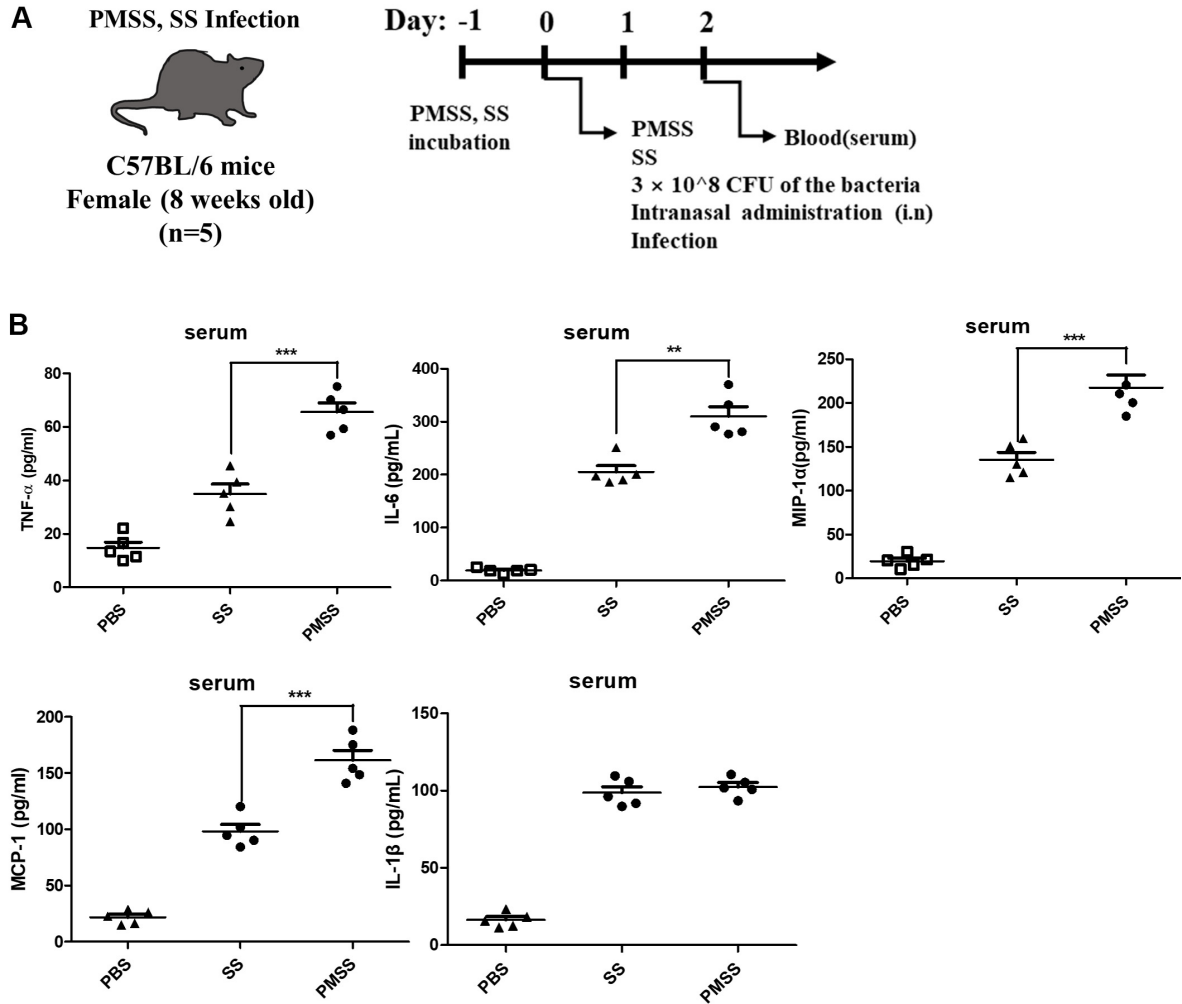
Quantification of pro-inflammatory cytokine levels in SS- and PMSS-infected mice. (**A**) Diagram outlining the experimental infection procedure. Eight-week-old female C57BL/6 mice were intranasally injected with 3 × 10^8^ CFU of either the SS or PMSS bacterial strains. (**B**) Serum levels of IL-6, TNF-α, IL-1β, MCP-1, and MIP-1α were quantified 24 h post-infection using commercially available ELISA kits. Each data point represents a single mouse. Group sizes were as follows: PBS (*n* = 5), SS (*n* = 5), and PMSS (*n* = 5). Statistical significance: *p < 0.05, **p < 0.01, ***p < 0.001.

Systemic inflammation in RA is associated with metabolic changes [8], and metabolites are known to contribute to the induction of inflammation [7]. In addition, SS and PMSS have been shown to induce inflammatory responses *in vitro* and *in vivo*, and PMSS is more infectious than SS. In addition, PMSS-infected cells show higher caspase-3/7 activity than SS-infected cells [4]. To determine whether SS and PMSS infection induce inflammatory responses in mouse serum (Fig. 5A), an inflammatory cytokine assay (ELISA) was performed. Although there was no significant difference in serum concentrations of IL-6 between the two group SS in PMSS-infected mice compared to SS-infected mice, the concentrations of TNF-α, IL-1β, MIP-1α, and MCP-1 were higher in PMSS-infected mice than in SS-infected mice (Fig. 5B). These results confirmed that SS and PMSS induce inflammatory responses in mice. In addition, PMSS induces a higher inflammatory response than SS, indicating the greater infectivity and inflammatory ability of PMSS.

### Pro-Inflammatory Effects of Selected Metabolites in MH-S Alveolar Macrophages

**Fig. 6:**
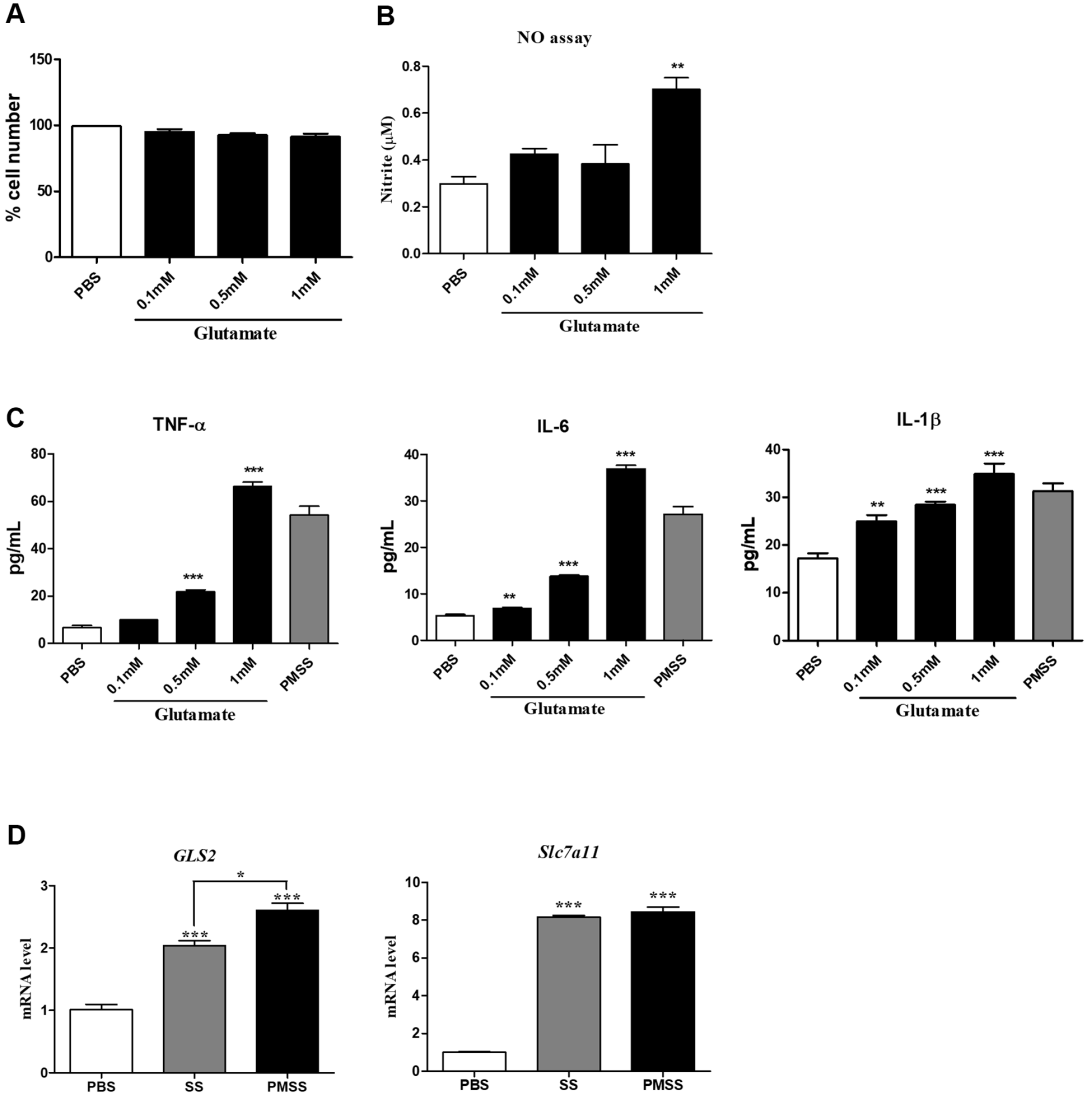
Pro-inflammatory effects of selected metabolites in MH-S alveolar macrophages. (**A**) Cell viability was determined by WST assay after treating MH-S cells (1 × 10^4^ cells/well) with varying concentrations of glutamate for 24 h. (**B**) Treatment with glutamate increased NO levels in the culture supernatant at 18 h in a dose-dependent manner. (**C**) Glutamate treatment significantly elevated IL-6, TNF-α, and IL-1β levels at 24 h, as measured by ELISA, in a dose-dependent manner. (**D**) PMSS-treated MH-S cells showed elevated mRNA expression of metabolite-associated genes: *GLS2*, *Slc7all* (Glutamateassociated genes). Statistical significance was assessed by Student’s *t*-test (*p* < 0.05, *p < 0.01, **p < 0.001).

Through our previous studies using cell cultures, 3D spheroid models, and mouse models, PMSS was found to have significantly stronger pro-inflammatory effects than *S. stutzeri* (SS). PMSS more potently induced the production of inflammatory cytokines and chemokines, activated the NF-κB and MAPK signaling pathways, and triggered both systemic and local inflammatory responses [4]. Based on untargeted serum metabolomic profiling, glutamate was identified among the top 20 upregulated metabolites in the serum of PMSS-infected mice. We evaluated the ability of glutamate to induce an inflammatory response in the alveolar macrophage cell line MH-S. We assessed MH-S cell viability after glutamate treatment up to 1 mM using WST analysis (Fig. 6A) and observed no cytotoxicity. Therefore, concentrations ≤1 mM were used in subsequent experiments. To assess its pro-inflammatory potential, nitric oxide (NO) production was measured in MH-S cells treated with glutamate at concentrations of 0, 0.1, 0.5, and 1 mM. NO production levels were elevated following glutamate treatment (Fig. 6B). This indicates that the upregulated metabolites associated with PMSS infection contribute to the inflammatory response. Glutamate treatment also significantly increased the secretion of the inflammatory cytokines TNF-α, IL-6, and IL-1β by the cells (Fig. 6C). In addition, to verify the inflammatory response, a positive control experiment using PMSS infection was conducted. To investigate the underlying mechanism, we examined the mRNA expression levels of genes associated with glutamate metabolism in MH-S cells. *GLS2*(Glutaminase 2) mediates the conversion of glutamine to glutamate [16, 17], *Slc7a11*(solute carrier family 7 member 11) functions in catalysis of glutamate efflux and cystine uptake as part of the cystine/glutamate antiporter system [18,19]. The expression of *GLS2* and *Slc7a11* was significantly elevated in the PMSS-treated group compared to the SS-treated group. PM-induced changes in metabolites contribute to inflammation (Fig. 6D). These findings demonstrate that glutamate, a metabolite upregulated in PMSS infection, directly contributes to the enhanced inflammatory response by increasing the expression of its related metabolic genes.

## Discussion

This study highlights the emerging recognition that PM is not merely a chemical pollutant but a complex mixture that includes biologically active constituents such as viable bacteria, which can significantly impact host physiology [20, 21]. While the chemical toxicity of PM has been well-documented [22, 23], our findings align with recent studies suggesting that microbial components including PMSS isolated from atmospheric fine dust may play a significant role in modulating host immune responses and contributing to disease pathology [24].

Our untargeted metabolomic analysis revealed that infection with PMSS induces extensive and distinct alterations in the host serum metabolome compared to infection with an environmental SS. Multivariate statistical analyses demonstrated a clear separation of metabolic profiles between the two group (Fig. 1), and volcano plots revealed a predominance of upregulated metabolites in PMSS-infected mice (Fig. 2), indicating strain-specific metabolic reprogramming. Further, random forest modeling and clustering analysis (Fig. 3) identified key discriminatory metabolites, reinforcing the unique metabolic signature elicited by PMSS infection.

Pathway enrichment analysis revealed that metabolites upregulated in the PMSS group were significantly associated with amino acid metabolism, particularly involving alanine, glutamate, and aspartate pathways, which are crucial for energy production and immune cell activation during inflammation [25, 26]. Moreover, activation of riboflavin metabolism points to enhanced redox activity and mitochondrial function, supporting sustained pro-inflammatory responses. Changes in butanoate metabolism, often linked to short-chain fatty acid signaling and the gut–lung axis, may further reflect a shift toward a host metabolic state that favors inflammation [27, 28]. Conversely, we observed suppression of CoQ10 and linoleic acid oxylipin biosynthesis pathways in PMSS-infected mice, which may imply an impairment in anti-inflammatory regulatory mechanisms. This dual effect activation of pro-inflammatory metabolic routes alongside suppression of anti-inflammatory pathways may contribute to the heightened and prolonged inflammatory state seen during PMSS infection.

Supporting these findings, elevated serum cytokines such as TNF-α, IL-6, MCP-1, and MIP-1α (Fig. 5) confirmed that PMSS infection triggers a more robust inflammatory response than SS. These findings have important implications for our understanding of microbe–host interactions within the context of air pollution. They demonstrate that the pathogenic potential of airborne bacteria such as PMSS extends beyond classical virulence factors to include the capacity to reprogram host metabolic networks in ways that exacerbate inflammation. This highlights an underappreciated mechanism whereby microbial components of particulate matter contribute to systemic immune activation through metabolite-driven signaling.

Overall, our results underscore the need to integrate microbial and metabolic perspectives into air pollution health risk assessments, particularly in light of the increasing recognition of PM as a biologically active entity [29]. From a broader perspective, these findings carry significant implications for public health. They suggest that the health risks of PM exposure may be underestimated if only chemical constituents are considered. The inclusion of microbial and metabolite profiling in air pollution assessments could lead to more accurate evaluations of disease risk and inform the development of targeted mitigation strategies. Furthermore, the identification of PMSS-specific metabolites as potential biomarkers of inflammation offers a promising avenue for early diagnosis or therapeutic intervention in PM-related disorders.

## Conclusion

This study demonstrates that PMSS, a bacterium isolated from particulate matter, actively reprograms host metabolism to induce systemic inflammation. PMSS infection upregulated pro-inflammatory metabolic pathways while suppressing anti-inflammatory circuits such as tryptophan metabolism, leading to elevated cytokine responses and metabolite-driven immune activation. These findings highlight the overlooked role of microbial components in PM as active contributors to inflammatory disease. By linking bacterial metabolites to host immune modulation, this work underscores the need to incorporate biological factors into air pollution research and opens new avenues for targeted interventions in PM-related health risks.

## Figures and Tables

**Table 1:** Top 50 up-regulated metabolites ranked by fold change and secretion abundance in SS/PMSS-infected mice.

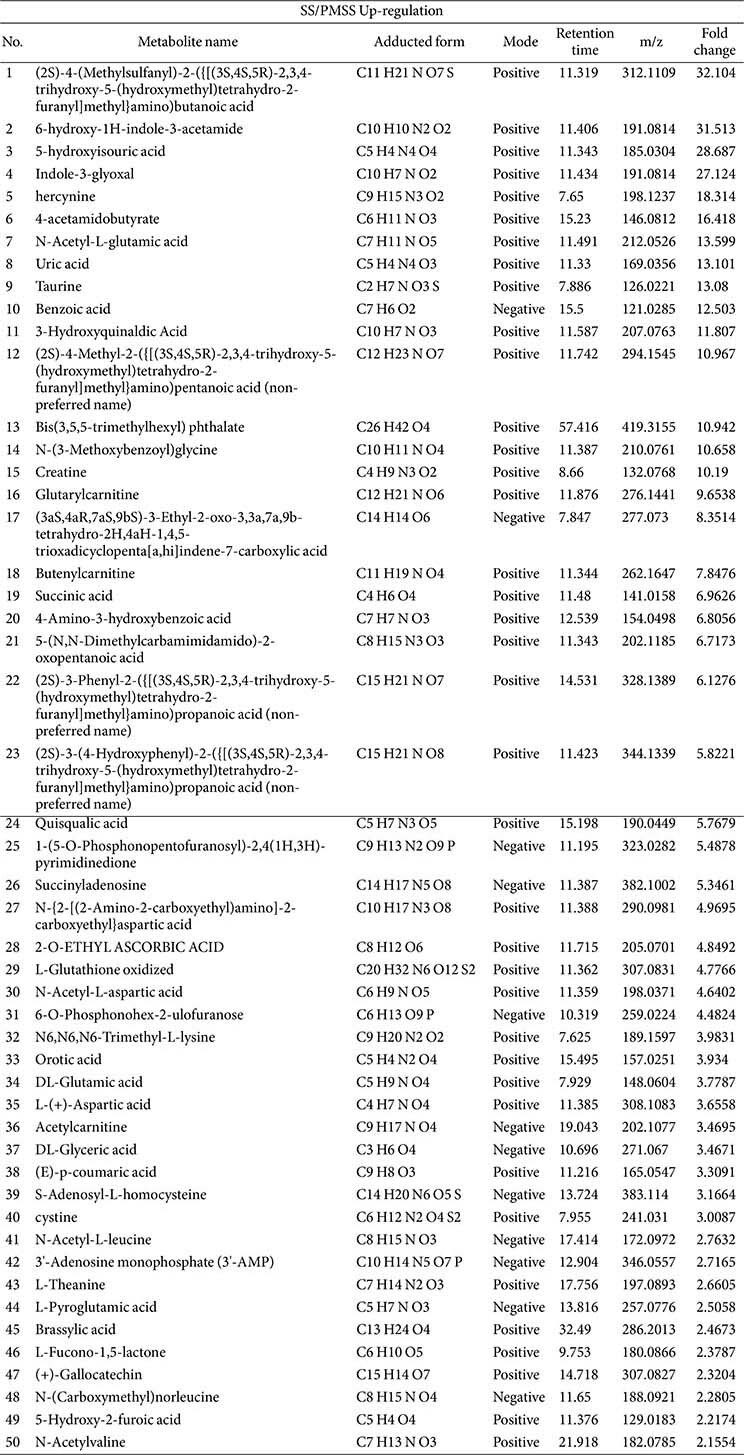

**Table 2:** Top 35 down-regulated metabolites ranked by fold change and secretion abundance in SS/PMSSinfected mice.

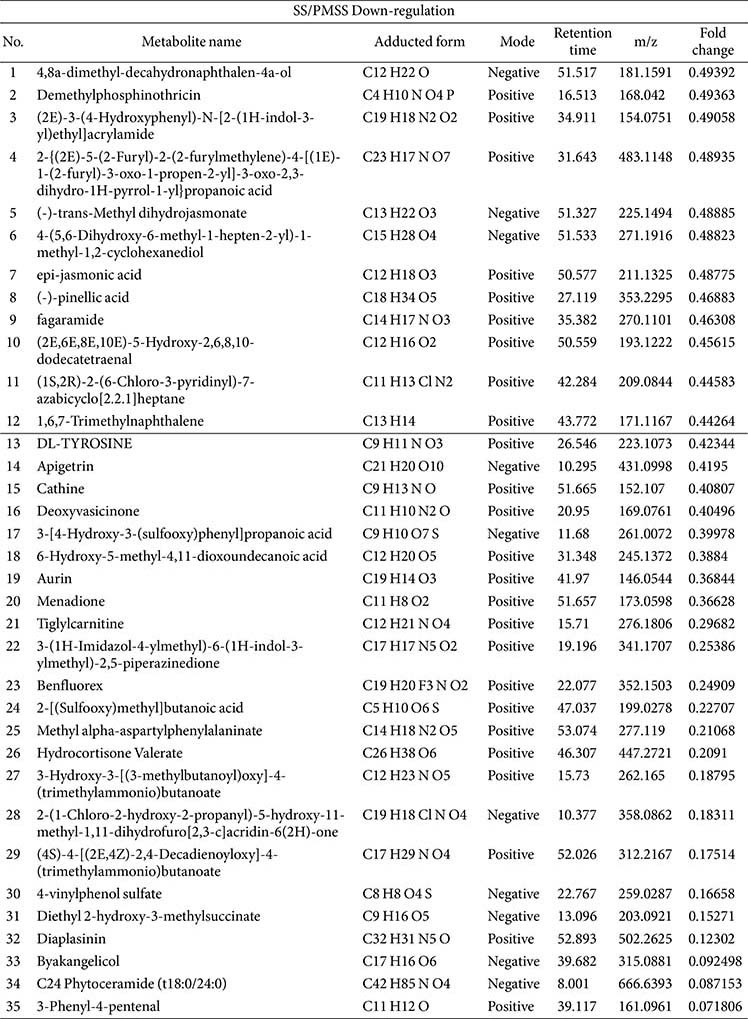

**Table 3:** Secreted metabolites from SS and PMSS revealed by untargeted metabolomic profiling.

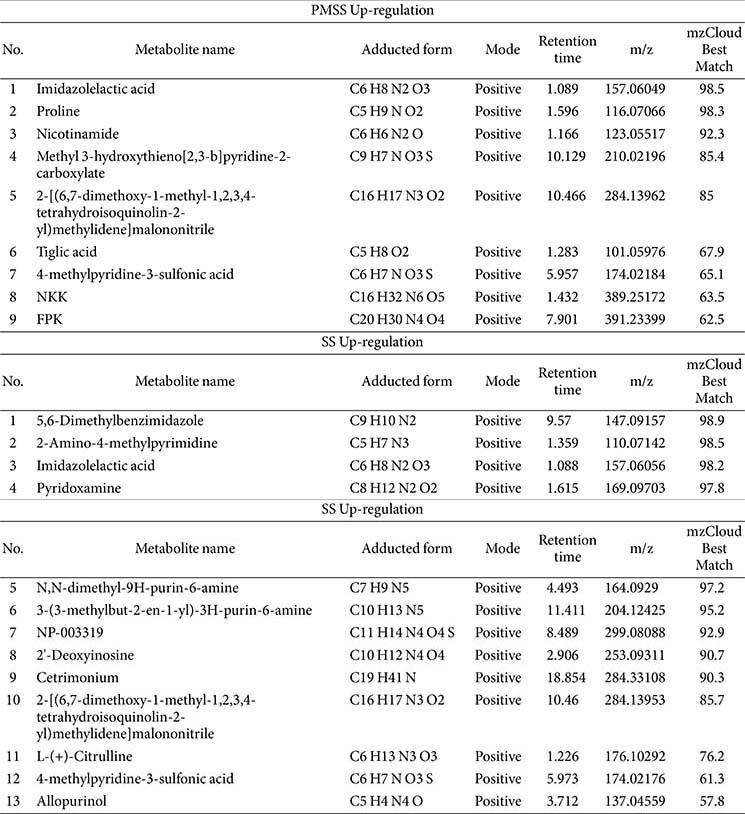

**Table 4:** RT-qPCR primer.